# A Gluten-Free Meal Produces a Lower Postprandial Thermogenic Response Compared to an Iso-Energetic/Macronutrient Whole Food or Processed Food Meal in Young Women: A Single-Blind Randomized Cross-Over Trial

**DOI:** 10.3390/nu12072035

**Published:** 2020-07-09

**Authors:** Brittney Dioneda, Margaret Healy, Maia Paul, Caitlin Sheridan, Alex E. Mohr, Paul J. Arciero

**Affiliations:** 1 Human Nutrition and Metabolism Laboratory, Department of Health and Human Physiological Sciences, Skidmore College, Saratoga Springs, NY 12866, USA; bdioneda@skidmore.edu (B.D.); mhealy1@skidmore.edu (M.H.); mpaul@skidmore.edu (M.P.); csherida@skidmore.edu (C.S.); 2 College of Health Solutions, Arizona State University, Phoenix, AZ 85281, USA; aemohr@asu.edu

**Keywords:** gluten-free, ultra-processed food, whole food, thermic effect of a meal, obesity

## Abstract

Consumption of ultra-processed food (PF) is associated with obesity risk compared with whole food (WF) intake. Less is known regarding the intake of gluten-free (GF) food products. The purpose of this study was to directly compare the thermic effect (TEM), substrate utilization, hunger/taste ratings, and glucose response of three different meals containing PF, WF, and GF food products in young healthy women. Eleven volunteers completed all three iso-caloric/macronutrient test meals in a single-blind, randomized crossover design: (1) whole food meal (WF); (2) processed food meal (PF); or (3) gluten-free meal (GF). TEM was significantly lower following GF compared with WF (−20.94 kcal/meal, [95% CI, −35.92 to −5.96], *p* = 0.008) and PF (mean difference: −14.94 kcal/meal, [95% CI, −29.92 to 0.04], *p* = 0.04), respectively. WF consumption resulted in significantly higher feelings of fullness compared to GF (mean difference: +14.36%, [95% CI, 3.41 to 25.32%], *p* = 0.011) and PF (mean difference: +16.81%, [95% CI, 5.62 to 28.01%], *p* = 0.004), respectively, and enhanced palatability (taste of meal) compared to PF meal (mean Δ: +27.41%, [95% CI, 5.53 to 49.30%], *p* = 0.048). No differences existed for substrate utilization and blood glucose response among trials. Consumption of a GF meal lowers postprandial thermogenesis compared to WF and PF meals and fullness ratings compared to a WF meal which may impact weight control and obesity risk over the long-term.

## 1. Introduction

A healthy diet continues to be the foundation of treating obesity and related conditions. Unfortunately, a shift towards a diet containing larger amounts of ultra-processed foods (PF) is consistently associated with increased risk of obesity and weight gain worldwide [1,2,3,4]. Broadly characterized, ultra (highly) PF include multi-ingredient industrial formulations such as packaged breads, sugar sweetened beverages, and processed cheese products [5]. The greater degree of food processing often includes added simple sugars, oils/fats, sodium, and less fiber than unprocessed whole food (WF) products [5]. Thus, PF may enhance palatability (taste) but reduce satiety due to reduced macromolecular complexity resulting in a lower thermic effect of a meal (TEM), which likely supports the higher risk of weight gain and obesity risk with higher PF intake [6,7]. The TEM is generally defined as the increase in metabolic rate that occurs following a meal. As such, it has a profound influence on modifying overall energy expenditure, and thus body weight control [8]. Recent data also confirm a metabolic risk of elevated insulin and glucose response to a PF compared to a WF meal, indicating a possible mechanism by which PF diets may increase type 2 diabetes risk [9]. However, to date, a direct comparison of the TEM difference between a WF meal versus a PF meal has been limited to one investigation [7].

Gluten-free (GF) food products are another form of PF rapidly gaining popularity for their proposed health benefits, especially for those with food intolerances or sensitivities [10,11,12], and are considered the first-line of treatment for those suffering from celiac disease [13]. Similar to highly processed gluten-containing grains, refined GF grains are chemically simple in molecular structure and easily metabolized, due to low fiber content and high glycemic index [14,15]. Interestingly, the majority of research on GF diet is among subjects with existing cardiovascular disease, demonstrating some [16,17], but not all [11,18], studies have reported a positive relation between GF intake and obesity risk, possibly due to a lower fiber, higher fat content and glycemic index of GF foods [19]. To our knowledge, no studies have directly compared a GF versus a PF or WF meal on TEM, hunger/fullness/palatability ratings and glucose response in healthy young women. Our laboratory previously demonstrated that a pancake meal containing resistant starch combined with protein more favorably enhances fat oxidation and feelings of fullness compared to a PF pancake meal challenge in a group of healthy women [20]. 

The elevated TEM and satiety induced by WF meals likely occur through certain common mechanisms [21]. Therefore, logic follows that WF meals may confer enhanced thermogenic, metabolic, and satiating benefits versus PF and GF meals. To address this issue, the current study systematically compared the TEM, satiety, and palatability ratings, and glucose response among iso-caloric/macronutrient WF, PF, and GF meals in a group of healthy young women. 

## 2. Materials and Methods

### 2.1. Participants

A total of twelve women from Saratoga Springs, NY were recruited for this study through email and flyers. Participants were non-smoking, healthy, young women with no known cardiovascular or metabolic diseases. Volunteers with a history of Celiac disease or other food allergies, metabolic disease or heart disease were excluded from this study. Participants with lactose intolerance/sensitivity were ineligible to participate in the study. To prevent possible influence on data, participants who engaged in excess use of dietary supplements were also excluded from the study. Because TEM is influenced by multiple factors, including meal size and frequency, macronutrient composition, age and physical activity level, only female participants with of similar age and meal consumption patterns were recruited. All participants provided written informed consent in adherence with the Skidmore College Human Subjects review board prior to participation, and the study was approved by the Human Subjects Institutional Review Board of Skidmore College: (IRB#: 1502-441). All experimental procedures were performed in accordance with the Federal Wide Assurance and related New York State regulations, which are consistent with the National Commission for the Protection of Human Subjects of Biomedical and Behavioral Research and in agreement with the Helsinki Declaration as revised in 1983. This trial was registered at clinicaltrials.gov as NCT04440826. 

### 2.2. Experimental Design

#### Study Timeline

**Figure 1:**
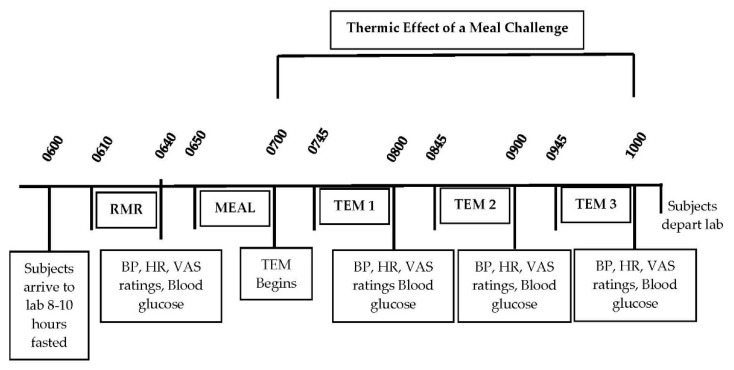
Timeline of Thermic Effect of a Meal (TEM) Test Days. RMR, resting metabolic rate; BP, blood pressure; HR, heart rate; VAS, visual analog scales for hunger, fullness, satiation, desire to eat.

All testing for the study occurred in the Human Nutrition and Metabolism Laboratory at Skidmore College during the spring of 2015. Prior to the first test meal challenge, participants’ body compositions were determined using the Life Measurements BODPod Body Composition Tracking System (Concord, CA). The day prior to each of the three test meal conditions, participants were instructed to keep a food record for 24-h prior to testing and asked to prepare and consume the same 24-h food record before each of the three testing days in order to standardize food intake the day before testing. The final evening dinner meal the night prior to each of the three test conditions was identical and consumed between 1800 and 2200 h, and was prepared by the participants using guidance from the investigators. All laboratory testing started between 0600 and 0800 h following a minimum 8-h fast (water was allowed) and at least 24-h restriction of caffeine and alcohol intake and vigorous physical activity. Specific test day timeline details are shown in Figure 1.

Upon arrival to the laboratory, body weight was measured (Befour Inc., Saukville, WI 53080 model number FS0900), followed by ~15 min of resting supine in a quiet, dimly lit room. Resting metabolic rate (RMR) was then measured for 30 min followed by a fasted blood pressure, heart rate, blood glucose, visual analog scales (VAS) of hunger, desire to eat, fullness/satiety, and palatability/taste of meal (see testing procedures below). Following the RMR, participants consumed one of three test meals (PF, WF, GF) within 10 min. Over the next 3 h following meal consumption, participants remained in a sedentary supine position while serial blood glucose, blood pressure and heart were obtained and VAS were completed (60, 120, and 180 min). Indirect calorimetry measured the thermic effect of each meal (TEM) (45–60, 105–120, 165–180 min). 

### 2.3. Grilled Cheese Test Meals

The study consisted of three separate visits to the Human Nutrition and Metabolism Laboratory, during which all participants consumed one of three grilled cheese test meals in a single-blind, randomized repeated measures crossover design: unprocessed whole foods (WF); ultra-processed foods (PF); or gluten-free foods (GF). Each test meal differed in degree of processing and each participant consumed each meal once, with a wash out period of at least 96 h between each trial. Each test meal was cooked on a non-stick griddle until golden brown and the cheese was melted. Because the meals were closely matched for total calories and macronutrients at the same time were made with ingredients to ensure they met the criteria for each meal type (i.e., WF, GF, and PF) some of the ingredients were different. All three meals were prepared following institutional guidelines using commercially available products and were isocaloric and contained similar macronutrient compositions as shown in Table 1.

## 3. Laboratory Testing Procedures

### 3.1. Resting Metabolic Rate (RMR), Thermic Effect of a Meal (TEM)

RMR was measured upon arrival using the ventilated hood system [22] with a computerized open-circuit indirect calorimeter (ParvoMedics Truemax 2400 Metabolic Measurement System, Salt Lake City, UT, USA). Following at least 15 min of quiet resting in a thermo-neutral (22–24 °C), semi-dark room, RMR was measured for 30 min in the supine position. Following RMR and other baseline measurements, participants underwent a thermic effect of a meal (TEM) challenge (PF, WF, GF) within 10 min and the meal order was randomized using a repeated measures crossover design. Following meal consumption, postprandial thermogenesis measurements occurred every 45 min for the next 180 min (45–60; 105–120; 165–180 min). The final 10 min of each 15 min measurement period (steady state) was used in the calculation of TEM (minutes 0–5 were discarded). TEM for the total 180 min was calculated using the average of each 10-min TEM measurement and multiplying it by 60 min (0–60; 61–120; 121–180 min) and the 180 min TEM value was the sum of each of the three 60 min TEM periods. The test–retest intraclass correlation (r) and coefficient of variation (CV) in *n* = 14 are: RMR (Kcal/min) *r* = 0.92, 4.2%, respectively.

### 3.2. Respiratory Exchange Ratio (RER) and Substrate Utilization

The RER and substrate utilization (carbohydrate and fat oxidation rates based on standardized caloric equivalents were also calculated from indirect calorimetry gas exchange as the volume of carbon dioxide produced to the volume of oxygen consumed (VCO_2_/VO^2^) (Parvomedics, Truemax 2400) and were calculated using the exact same method described above for 180 min TEM. A 180 min TEM was chosen to capture the majority of the postprandial response. The total kilocalories eaten for each of the 3 test meals were iso-caloric and similar in macronutrient composition (Table 1), which allowed for the direct comparison of differences in the degree of food processing on the thermogenic response. 

### 3.3. Feelings of Fullness, Satiety, Hunger, Desire to Eat, and Palatability (Taste)

Fullness, satiety, hunger, and desire to eat were all evaluated using a 100-mm visual analog scale (VAS). Participants were prompted with four questions regarding fullness, satiety, hunger, and desire to eat and then asked to draw a vertical line on the VAS scale representing how they felt at that moment. A vertical line mark at 0 mm represented no feelings, whereas a mark at 100 mm indicated extreme feelings. The degree to which each sensation was felt was quantified by measuring how far the mark was from the 0 mm point. A standard millimeter ruler was used for all measurements and all scores were computed by the same investigator. VAS scales were completed during each of the three test meal conditions at baseline (RMR) and every hour during the TEM meal challenge (60, 120, and 180 min). In addition, immediately after consuming each meal, the palatability (taste quality) was evaluated using a 1–10 scale, in which 1 was the worst tasting ‘grilled cheese’ meal and 10 was the best tasting ‘grilled cheese’ meal. 

### 3.4. Blood Glucose

Blood glucose was analyzed following the RMR and every hour after meal ingestion (TEM; 60, 120, 180 min) via finger stick with a OneTouch Blood Glucose Analyzer (LifeScan IP Holdings, LLC., Malvern, PA, USA).

### 3.5. Heart Rate and Blood Pressure

Resting heart rate and blood pressure were manually recorded in the supine position as previously described [23]. Heart rate was obtained by palpation from the radial pulse for 1 min and blood pressure measurements were obtained using a sphygmomanometer (American Diagnostic Corp., 55 Commerce Drive, Hauppauge NY 11788, USA) and stethoscope (Littman Quality, St. Paul, MN 55144, USA) by a trained investigator on each of the three test meal days following the RMR measurement.

### 3.6. Statistical Data Analysis

Normality statistics (Shapiro–Wilk’s tests and skewness and kurtosis z-scores) and probability plots (Q-Q plots and histograms) were generated to test normality assumptions, and log transformations were performed as appropriate. Area under the curve (AUC), absolute and percent changes were conducted as described previously [20,24]. Briefly, blood glucose and TEM area under curve (AUC) were calculated by the trapezoidal rule for the entire 3-h postprandial period as an additional evaluative method. Absolute changes were calculated as the average baseline value subtracted from each post-meal value. Percent changes were calculated as the delta between baseline and each post mealtime point divided by the baseline value. To determine the effect of meal type on the outcome variables, linear mixed-effect models were employed with a random intercept for subject, and time and meal type as fixed factors. Additionally, to assess whether the effect of meal type was different between time points, an interaction term (meal*time) was added to the model to provide unbiased estimates of time and treatment effects under a missing-at-random assumption. Relevant covariates (i.e., BMI, age, and fasting glucose concentration) were added to the model to adjust for possible confounding. Multiple comparisons were made on generated estimated marginal means for both main effects (time and meal) and interaction effects (time*meal) with Bonferroni post-hoc tests. Power analysis and sample size was based on previous research comparing a WF vs. PF meal on diet induced thermogenesis [7]. Based on an estimated effect size of *F* = 0.65, with power = 0.80 and α-level at 0.05, we estimated a minimal total sample size of 9 participants to detect a significant time*meal effect in postprandial energy expenditure. All analyses were performed using SPSS 26.0 for Windows (SPSS Inc., Armonk, New York 10504-1722, USA). An α-level was set at a significance of *p* < 0.05. Data are shown as mean values (with 95% CIs) unless otherwise noted.

## 4. Results

### 4.1. Participants and Compliance

One participant did not complete all three testing conditions and was not included in the data analysis. Baseline physical characteristics of the 11 subjects who completed all testing are presented in Table 2.

### 4.2. Resting Metabolic Rate (RMR) and Thermic Effect of a Meal (TEM)

**Figure 2:**
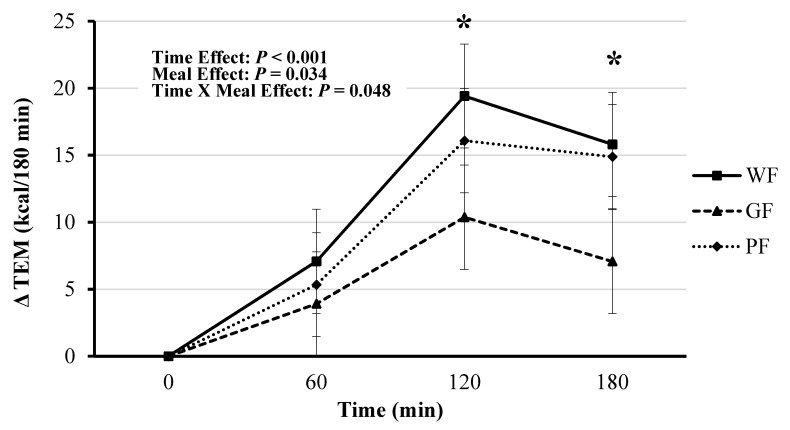
Comparison of postprandial thermogenic responses (TEM) to whole foods (WF), gluten-free (GF) and processed foods (PF) meals calculated as incremental area under the curve (AUC) for 0–60, 60–120, and 120–180-min periods. * Significance between AUC for WF and PF meals compared to the GF meal (*p* < 0.05). Values displayed as means ±95% confidence intervals.

RMR was similar among all three meal trials (Table 3 and Table S1). The TEM was significant for time (*F*(3, 121) = 32.81, *p* < 0.001), meal (*F*(2, 121) = 3.47, *p* = 0.034), and meal*time (*F*(6, 121) = 2.21, *p* = 0.048). The GF test meal had lower overall TEM compared to WF (mean difference: −0.055 kcal/min, [95% CI, −0.086 to −0.019], *p* = 0.002) and PF (mean difference: −0.041 kcal/min, [95% CI, −0.075 to −0.008], *p* = 0.022). The GF demonstrated lower TEM at 120 min compared to WF (Bonferroni post hoc analysis, mean Δ: −0.069 kcal/min, [95% CI, −0.136 to −0.002], *p* = 0.044) and PF (mean Δ: −0.085 kcal/min, [95% CI, −0.152 to −0.018], *p* = 0.014; Table 3 and Figure 2).

**Figure 3:**
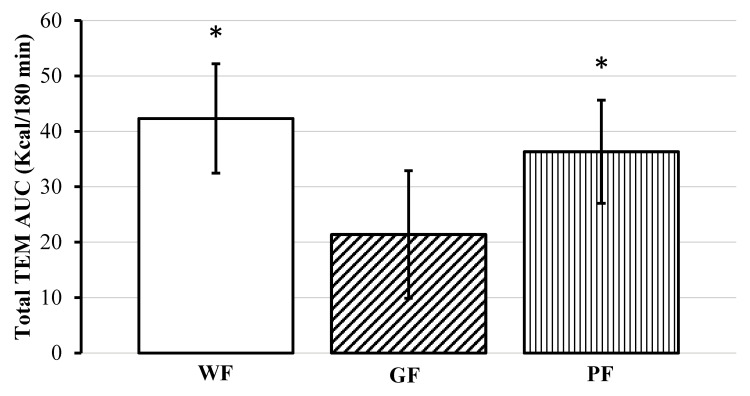
Comparison of total thermic effect of meal (TEM) area under the curve (AUC) for the 180–minute postprandial period following after consumption of whole foods (WF), gluten-free (GF) and processed (PF) meals. * Significant difference compared to GF *p* < 0.05. Data displayed as means ±95% confidence intervals.

This trend continued at 180 min, but only compared to WF meal (mean Δ: −0.079 kcal/min, [95% CI, −0.146 to −0.012], *p* = 0.021). Absolute AUC for TEM was significant (*F*(2, 22) = 4.46, *p* = 0.024), with GF having a lower amount of calories burned compared to WF (mean difference: −20.94 kcal/meal, [95% CI, −35.92 to −5.96], *p* = 0.008) and PF (mean difference: −14.94 kcal/meal, [95% CI, −29.92 to 0.04], *p* = 0.04) (Figure 3).

### 4.3. Respiratory Exchange Ratio (RER) and Substrate Utilization

**Figure 4:**
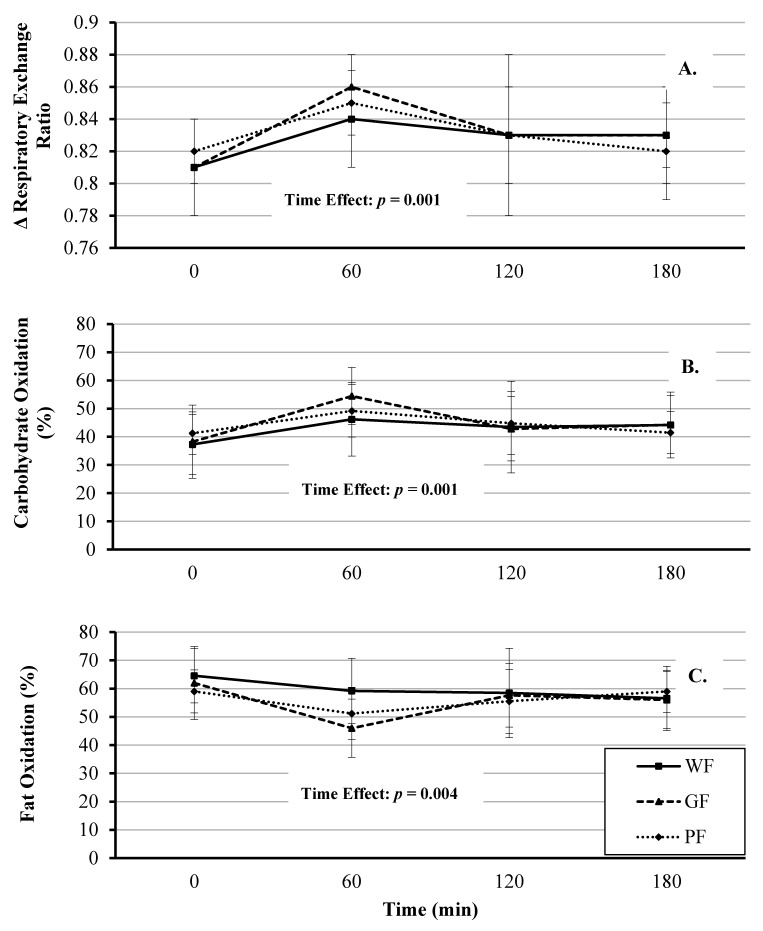
Comparison of respiratory exchange ratio (Panel **A**), carbohydrate (Panel **B**), and fat (Panel **C**) oxidation responses after the consumption of whole foods (WF), gluten-free (GF) and processed (PF) meals. Significant time effect for all three conditions (*p* < 0.05). Values are displayed as means ±95% confidence intervals.

There was a significant fixed effect of time on RER (*F*(3, 121) = 5.68, *p* = 0.001), with significant mean increases occurring at 60 min compared to baseline (mean Δ: +0.032 [95% CI, 0.011 to 0.054], *p* = 0.001; Figure 4 Panel A, Table S1). The fixed effects of meal and meal*time were not significant (*F*(2, 121) = 0.396, *p* = 0.674 and *F*(6, 121) = 0.523, *p* = 0.79, respectively).

The percent change in carbohydrate oxidation over time was significant (*F*(3, 121) = 6.47, *p* < 0.001), with a significant increase at 60 min compared to baseline (mean Δ: +11.00%, [95% CI, 6.02 to 15.98%], *p* < 0.001). The effect of meal and meal*time were not significant (*F*(2, 121) = 1.57, *p* = 0.21 and *F*(6, 121) = 0.72, *p* = 0.632, respectively). Similarly, the percent change in fat oxidation was significant (*F*(3, 121) = 4.73, *p* = 0.004), with a significant decrease occurring at 60 min compared to baseline (mean Δ: −9.73%, [95% CI, −14.84 to −4.62%], *p* < 0.001). The effect of meal and meal*time were not significant (*F*(2, 121) = 1.43, *p* = 0.24 and *F*(6, 121) = 1.19, *p* = 0.31, respectively). Comparison of substrate utilization between meals is presented in Figure 4 (Panels B and C) and Table S1.

### 4.4. Feelings of Fullness, Satiety, Hunger, Desire to Eat, and Palatability (Taste)

There was a significant effect of time (*F*(3, 33) = 28.34, *p* < 0.001), and meal for the feeling of fullness (*F*(2, 88) = 5.28, *p* < 0.001), with WF meal eliciting greater fullness compared to both GF (mean difference: +14.36%, [95% CI, 3.41 to 25.32%], *p* = 0.011) and PF (mean difference: +16.81%, [95% CI, 5.62 to 28.01%], *p* = 0.004) (Table 4). 

Interestingly, there was no significant interaction effect of meal*time (*F*(6, 88) = 0.85, *p* = 0.535). The feelings of hunger and desire to eat were all significantly decreased over time (*F*(3, 121) = 20.55 to 21.58, all *p*’s < 0.001) at 60, 120, and 180 min following meal ingestion (Table 4). However, the fixed effects of meal and meal*time were not significant (*F*(2, 121) = 0.165 to 1.61, all *p*’s ≥ 0.20 and *F*(6, 121) = 0.344 to 1.978, all *p*’s ≥ 0.077, respectively). There was a non-significant trend for desire to eat (meal*time; *p* = 0.077), with Bonferroni post hoc analysis showing a trend for increased values for PF compared to WF at 120 and 180 min (mean Δ: +26.42%, [95% CI, 0.65 to 53.49%; *p* = 0.056, and mean Δ: +16.62%, [95% CI, 6.55 to 26.69%], *p* = 0.08, respectively) (Table 4).

Palatability showed a significant effect of meal type (*F*(2,22) = 3.52, *p* = 0.047), with WF meal showing greater meal enjoyment (taste) rating than PF meal (mean Δ: +27.41%, [95% CI, 5.53 to 49.30%], *p* = 0.048; Table 5). 

### 4.5. Blood Glucose Response

**Figure 5:**
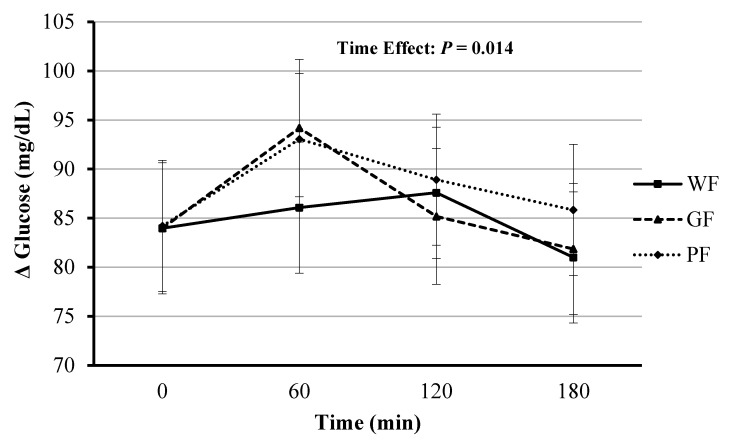
Comparison of blood glucose responses after the consumption of whole foods (WF), gluten-free (GF) and processed (PF) meals. Significant time effect for all three conditions (*p* < 0.05). Values displayed as means ±95% confidence intervals.

The blood glucose main effect of time was significant (*F*(3, 33.17) = 4.13, *p* = 0.014), with significant changes occurring at 180 min compared at 60 min in the postprandial period (mean Δ: −8.20 mg/dL [95% CI, −15.38 to −1.02], *p* = 0.018). The fixed effects of meal type and meal*time were not significant (*F*(2, 87.65) = 1.49, *p* = 0.229 and *F*(6, 87.63) = 0.81, *p* = 0.569, respectively). Blood glucose AUC did not differ significantly between the three meals (*F*(2, 22) = 0.82, *p* = 0.455; Figure 5).

## 5. Discussion

The primary aim of this study was to systematically compare the thermic response (TEM), blood glucose and fullness, hunger, satiety, and palatability ratings among three iso-caloric/macronutrient grilled cheese meals differing only in degree of processing in healthy young women. The main findings of the current study reveal that a gluten-free (GF) meal is: (1) significantly (*p* < 0.01) more thermogenic efficient compared to ultra-processed (PF; −41%) and unprocessed whole food (WF; −50%) meals, respectively; (2) WF meal consumption resulted in significantly (*p* < 0.01) greater feelings of fullness compared to GF (+14.36%) and PF (+16.81%), respectively, and enhanced (*p* < 0.05) palatability (taste of meal) compared to PF meal (+27.41%); and (3) there was a strong trend (*p* = 0.077) for increased desire to eat following the PF compared to WF at 120 (+26.42%) and 180 (16.62%), respectively. 

Taken together, the current findings demonstrate that an acute GF meal challenge produces a significantly lower thermogenic response (calorie burn) compared to WF and PF meals of equal caloric and macronutrient composition. Furthermore, a WF meal results in a greater sensation of fullness compared to both PF and GF and enhanced taste compared to a PF meal. These findings may have substantial public health implications regarding how specific dietary advice is recommended based on food processing techniques for both weight control and obesity risk. 

### 5.1. Thermic Effect of a Meal (TEM)

The GF meal induced a significantly lower thermogenic response compared to both PF and WF meals, whereas PF and WF were similar to each other. Our finding contradicts a previous study showing that a PF meal produced a 47% lower TEM compared to a WF meal [7]. The authors noted that protein content was 5% lower in their PF meal and this may have contributed to the ‘metabolic disadvantage’ of the PF meal. In the present study, all three meals contained equal amounts of macronutrients, including protein (16–17 g), and therefore some other factor(s) are likely responsible for the similar metabolic advantage (higher TEM) of WF and PF compared to GF meal. One of the most interesting findings of the current study was the similar higher TEM response of WF and PF compared to GF. Although it is beyond the scope of this study, it is interesting to speculate whether the glycemic index (GI) of the meals may have influenced the TEM response. Research is mixed whether varying GI levels of a meal influence TEM [25,26]. In addition, the elimination of gluten from the diet also removes the beneficial pre- and pro-biotic action gluten plays in the gut microbiota, including reductions in fiber and fructans [27]. Over time, this dramatic reduction in TEM with GF diets may result in significant weight gain. Although the lack of gut microbiota measurements in the current study precludes our ability to draw firm conclusions, this concept clearly warrants further investigation regarding the “metabolic and thermogenic disadvantage” of a GF diet. 

### 5.2. Respiratory Exchange Ratio (RER) and Substrate Utilization

As expected, the current study showed a significant increase in RER following all three meals and there was no difference among the trials. To our knowledge, no study has directly compared the effects of food processing utilizing meal challenges of WF, PF, and GF foods on postprandial substrate utilization. It is well-accepted that diets rich in ultra PF are more obesogenic due to increased energy density, added sugars, and fats, as well as nutrient-depleted of micronutrients, bioactives, and fiber [28,29]. Based on this data, it is hypothesized that PF meals (including GF meals) would increase RER via higher carbohydrate oxidation and lower fat oxidation and therefore alter the TEM response. However, some other mechanism(s) is responsible, as our data do not support this. Previous work from our laboratory found that a pancake meal containing resistant starch with and without protein increased fat oxidation compared to a processed pancake meal [20]. By design, it is important to highlight, the current study closely matched the calories and macronutrient content of all three meals, including sugar content. The degree to which different types of sugars, including the glycemic index, and fiber differences (6 vs. 2 g) impacted the results remains to be determined and offers an interesting area of follow-up study. 

### 5.3. Feelings of Fullness, Satiety, Hunger, Desire to Eat, and Palatability (Taste)

The WF test meal increased feelings of fullness compared to PF and GF meals, and showed a strong trend for reduced ‘desire to eat’ versus the PF test meal. These data are consistent with previous findings [7], supporting the well-documented satiating effects of un-processed/refined WF [30], and the significantly higher palatability (taste) of the WF compared to the PF meal response observed in the current study. The current study findings are partially in support of the landmark research by LeBlanc and Brondel [31] showing more palatable meals elicit increased postprandial thermogenic (TEM) responses versus tasteless unpalatable meals containing similar ingredients. Although the PF meal in the current study was not necessarily ‘tasteless’ or ‘unpalatable, it remains unclear as to why the WF meal showed significantly enhanced palatability but similar TEM response to the PF meal, and suggests some other, yet defined, mechanism may be responsible. From a public health perspective, it is clear that a WF diet encourages feelings of fullness (satiety) and palatability, and increases postprandial thermogenesis for optimal weight control compared to PF and GF diets. 

### 5.4. Blood Glucose Response

The current study showed no differences among test meals on blood glucose response, suggesting that degree of processing had little influence on blood glucose. Other research studies on acute meal ingestion show both similar [20,32,33] and conflicting [9] blood glucose responses to varying processed meals. Most previous studies reporting lower glucose responses to meal ingestion used test meals with varying amounts of fiber, protein, or fat [34]. The degree to which gastro-entero-hepatic hormones, such as CCK, GLP-1, GIP, PYY and ghrelin, may be involved in the glucose (and insulin) response warrants further study, especially in light of the potential impact of the gut microbiota playing a role. There is strong agreement that ultra-processing disrupts the physical structure of intact food, making the macromolecular structure more simple, and thus easier and faster to digest, resulting in more rapid increases in plasma glucose and insulin [35]. The current study does not support either PF or GF meals adversely affecting glycemic control compared to a WF meal in young healthy women.

### 5.5. Limitations

Several limitations were present in the current study. The total number of participants was small and the age range was narrow, both of which minimize the application of our findings to men and other age ranges. Second, the current study did not include measures of the gut microbiota, gastro-entero-hepatic hormones, including insulin, and thus cannot draw any conclusions regarding the influence of these factors on the findings. In addition, the glycemic index and load of each of the test meals was not available, and therefore the degree that these factors influenced the TEM and/or hunger ratings is unknown. The influence that glycemic index and load may have had on the results is likely minimal given the similar TEM of PF to WF, and the likely similar glycemic index of the GF and PF meal. This area warrants further investigation.

## 6. Conclusions

In conclusion, the current study found that the degree of food processing impacts postprandial thermogenesis and subjective feelings of fullness, satiation and palatability. Specifically, a GF meal induces a lower thermogenic response than an iso-caloric/macronutrient WF and PF meal. In addition, the WF grilled cheese meal provided greater feelings of fullness compared to GF and PF, as well as enhanced palatability (taste of meal) compared to PF meal. Interestingly, an ultra PF meal tended to increase desire to eat soon after meal consumption compared to the WF meal. The magnitude of these findings is biologically relevant and may have important public health implications for body weight control if sustained over the long-term.

## Figures and Tables

**Table 1:** Nutritional analysis and ingredients of the three test meals.

Nutritional Analysis	WF	PF	GF
Energy (kcal)	580	587	580
Total carbohydrate (g)	67	67	64
Total sugar (g)	36	39	34
Total fat (g)	26.5	28.5	31
Total protein (g)	17.6	16	16
Total fiber (g)	6	2	8
**Ingredients**			
Bread	Ezekiel Bread, 68 g	Wonder bread, 63 g	Rudi’s Gluten-Free Bread, 68 g
Butter	Kate’s of Maine Butter, 14 g	I Can’t Believe It’s Not Butter, 14 g	I Can’t Believe It’s Not Butter, 14 g
Cheese	Cabot Vermont Cheddar, 28 g	Classic American Kraft Singles, 36 g	Cabot Vermont Cheddar, 28 g
Drink	Bolthouse Fruit Juice, 336 g	Fanta Orange Soda, 258 g	Ocean Spray Cranberry Juice, 384 g

WF: Whole food; PF: Processed food; GF: Gluten free.

**Table 2:** Subject characteristics ^a^.

*N*	11		
Age (years)	20.63	±	1.24
Weight (kg)	62.38	±	8.04
Height (cm)	166.93	±	7.64
BMI	22.34	±	2.23
Percent fat mass (%)	26.02	±	5.08
Systolic BP (mmHg)	103.71	±	11.83
Diastolic BP (mmHg)	65.51	±	7.89
Resting HR (bpm)	61.60	±	10.38

^a^ Data presented as mean ± standard deviation.

**Table 3:** Calories consumed and postprandial response for thermic effect of the three test meals ^a^.

Meal	Kcal	RMR ^b^	95% CI	60 m TEM ^c^	95% CI	120 m TEM	95% CI	180 m TEM	95% CI	Total TEM ^d^	95% CI
WF	580	1800	1724–1875	7.08	3.19–10.97	19.42 *	15.54–23.31	15.81 *	11.93–19.69	42.32 *	31.85–52.78
GF	587	1724	1657–1791	3.92	0.04–7.80	10.38	6.49–14.27	7.07	3.19–10.96	21.38	10.91–31.84
PF	580	1783	1709–1857	5.34	1.46–9.23	16.09 *	12.20–19.97	14.89 *	11.01–18.78	36.32 *	25.86–46.78

^a^ Effects based on estimated marginal means. Bonferroni adjustments were conducted for multiple comparisons. ^b^ RMR: Resting metabolic rate taken at baseline. ^c^ TEM: Thermic effect of meal values calculated as incremental area under the curve for 0–60, 60–120, and 120–180-min periods postprandially. ^d^ Total area under the curve calculated for the 0–180-min period. WF: Whole food; GF: Gluten free; PF: Processed food. * Significant difference compared to GF *p* < 0.05.

**Table 4:** Effects of meal type on mean values of hunger and satiety rating outcome measurements at baseline and 60, 120, and 180 min in the postprandial period ^a^.

		Baseline		60 min		120 min		180 min	
Outcome Variable	Meal	Mean	95% CI	Mean	95% CI	Mean	95% CI	Mean	95% CI
Fullness (mm)	WF *	37.55	27.49–47.59	60.00	49.95–70.05	62.64	52.59–72.68	58.36	48.32–68.41
	GF	23.09	13.04–33.14	59.18	49.13–69.23	57.73	47.68–67.78	51.09	41.04–61.14
	PF	30.82	20.77–40.87	53.27	43.22–63.32	54.00	43.95–64.05	49.00	38.95–59.05
Hunger (mm)	WF	56.46	45.79–67.12	27.91	17.25–38.57	28.73	18.06–39.39	34.55	23.88–45.21
	GF	52.82	42.15–63.48	28.00	17.34–38.66	30.82	20.15–41.48	30.91	20.25–41.48
	PF	53.27	42.61–63.94	32.82	22.15–43.48	38.73	28.06–49.39	38.46	27.79–49.12
Desire to Eat (mm)	WF	58.27	47.29–69.26	27.91	16.93–38.89	32.00	21.02–42.98	37.18	26.19–48.16
	GF	60.64	49.65–71.62	27.91	16.93–38.89	33.64	22.65–44.62	36.91	25.93–47.89
	PF	50.64	39.65–61.62	30.55	19.56–41.53	40.46	29.47–51.44	43.09	32.11–54.07
Satiety (mm)	WF	58.64	48.42–68.86	36.36	26.14–46.59	41.27	31.05–51.49	47.64	37.42–57.86
	GF	60.55	50.32–70.77	40.09	29.87–50.31	43.27	33.05–53.49	45.36	35.14–55.58
	PF	56.18	45.96–66.40	37.55	27.32–47.77	44.09	33.87–54.31	48.46	38.23–58.68

^a^ Effects based on estimated marginal means. Bonferroni adjustments were conducted for multiple comparisons. WF: Whole food; GF: Gluten free; PF: Processed food. * Significant main effect of meal; increased fullness vs. GF and PF, *p* < 0.05.

**Table 5:** Palatability ratings of the whole foods, processed and gluten-free meals.

	WF	PF	GF
Palatability	7.18 [6.15–8.21] *	5.63 [4.54–6.73]	6.68 [5.62–7.75]

Values displayed as means ± 95% CI], * significance between WF and PF (*p* < 0.05).

## Supplementary Materials

The following are available online at https://www.mdpi.com/2072-6643/12/7/2035/s1, Table S1: Effects of meal type on mean values of metabolic outcome measurements and blood glucose at baseline and 60, 120, and 180 min in the postprandial period.

## Abbreviations

The following abbreviations are used in this manuscript:

PF	processed food
WF	whole food
GF	gluten-free food
RMR	resting metabolic rate
TEM	thermic effect of a meal
BMI	body mass index
RER	respiratory exchange ratio
AUC	area under the curve
VAS	visual analog scale
