# Supplementary material for: A Gluten-Free Meal Produces a Lower Postprandial Thermogenic Response Compared to an Iso-Energetic/Macronutrient Whole Food or Processed Food Meal in Young Women: A Single-Blind Randomized Cross-Over Trial
Source: Nutrients. 2020 Jul 9;12(7):2035. doi: 10.3390/nu12072035 (PMC7400113; doi:10.3390/nu12072035)
Supplement: Supplementary file 1 [file nutrients-12-02035-s001.pdf]

Table S1. Effects of meal type on mean values of metabolic outcome measurements and blood glucose at baseline and 60, 120, and 180 minutes in the postprandial period.[a]

		Baseline		60 min		120 min		180 min	
**Outcome Variable**	**Meal**	**Mean**	**95% CI**	**Mean**	**95% CI**	**Mean**	**95% CI**	**Mean**	**95% CI**
RMR & TEM (kcal/minute)[b]	WF	1.09	1.00-1.19	1.33	1.24-1.42	1.28*	1.19-1.37	1.28*	1.18-1.37
	GF	1.12	1.02-1.21	1.27	1.12-1.36	1.21	1.12-1.30	1.19	1.15-1.29
	PF	1.11	1.01-1.21	1.29	1.20-1.39	1.29*	1.20-1.39	1.25	1.16-1.34
RER	WF	0.81	0.78-0.84	0.84	0.81-0.86	0.83	0.80-0.86	0.83	0.80-0.86
	GF	0.81	0.78-0.85	0.86	0.84-0.89	0.83	0.80-0.85	0.83	0.81-0.86
	PF	0.82	0.80-0.84	0.85	0.82-0.87	0.83	0.81-0.86	0.82	0.79-0.85
Carbohydrate Oxidation (%)	WF	37.27	27.62-46.93	46.18	36.53-55.84	43.46	33.79-53.11	44.18	34.53-53.84
	GF	38.27	28.62-47.93	54.46	44.79-64.11	42.82	33.16-52.48	44.27	34.62-53.93
	PF	41.27	31.62-50.93	49.18	39.53-58.84	44.82	35.15-54.47	41.46	31.79-51.11
Fat Oxidation (%)	WF	64.55	55.19-73.89	59.18	49.83-68.54	58.45	49.10-67.81	56.54	47.19-65.89
	GF	62.00	52.65-71.35	46.00	36.65-55.35	57.64	48.28-66.99	56.00	46.65-65.35
	PF	59.00	49.65-68.35	51.18	41.83-60.54	55.55	46.19-64.89	59.00	49.64-68.34
Glucose (mg/dL)	WF	83.97	77.29-90.65	86.07	79.39-92.75	87.59	80.91-94.27	81.00	74.32-87.68
	GF	83.97	77.29-90.65	94.18	87.51-100.86	85.17	78.25-92.09	81.86	75.19-88.54
	PF	84.21	77.53-90.89	93.05	86.37-99.72	88.91	82.23-95.59	85.83	79.15-92.50

[a]Effects based on estimated marginal means. Bonferroni adjustments were conducted for multiple comparisons.
[b]*RMR:* Resting metabolic rate taken at baseline. *TEM*: Thermic effect of meal taken at 60, 120, and 180 minutes postprandially.
WF: Whole food; *GF*: Gluten free; *PF*: Processed food; *RER*: Respiratory exchange ratio.
*Significant difference compared to GF $P < 0.05$
